# Knockdown of SNHG14 Alleviates MPP^+^-Induced Injury in the Cell Model of Parkinson’s Disease by Targeting the miR-214-3p/KLF4 Axis

**DOI:** 10.3389/fnins.2020.00930

**Published:** 2020-09-21

**Authors:** Shufang Zhou, Dan Zhang, Junnan Guo, Junshi Zhang, Yong Chen

**Affiliations:** ^1^Department of Neurology, Huaihe Hospital of Henan University, Kaifeng, China; ^2^Department of Dentistry, Huaihe Hospital of Henan University, Kaifeng, China

**Keywords:** Parkinson’s disease, SNHG14, miR-214-3p, KLF4, cytotoxicity

## Abstract

**Background:**

Parkinson’s disease (PD) is the second most common neurodegenerative disease. Long non-coding RNA (lncRNA) small nucleolar RNA host gene 14 (SNHG14) has been demonstrated as an important regulator in PD pathology. However, the functional mechanisms played by SNHG14 in PD remain largely unclear.

**Methods:**

We used 1-methyl-4-phenyl-1,2,3,6-tetrahydropyridine (MPTP) and 1-methyl-4-phenylpyridinium (MPP^+^) to establish PD mouse and cell models. The levels of SNHG14, miR-214-3p, and Krüppel-like factor 4 (KLF4) were gauged by quantitative real-time polymerase chain reaction (qRT-PCR) or western blot analysis. Cell viability and apoptosis were determined using the Cell Counting-8 Kit (CCK-8) assay and flow cytometry, respectively. The levels of inflammatory cytokines were evaluated by ELISA. The relationships among SNHG14, miR-214-3p, and KLF4 were confirmed by dual-luciferase reporter and RNA immunoprecipitation (RIP) assays.

**Results:**

Our data indicated that SNHG14 was upregulated and miR-214-3p was downregulated in PD models. SNHG14 knockdown ameliorated MPP^+^-stimulated damage in SK-N-SH cells, as evidenced by the enhancement in cell viability and the suppression in cell apoptosis and pro-inflammatory cytokine production. Mechanistically, SNHG14 directly targeted miR-214-3p via binding to miR-214-3p, and SNHG14 knockdown protected SK-N-SH cell from MPP^+^-stimulated cytotoxicity by upregulating miR-214-3p. KLF4 was a direct target of miR-214-3p, and SNHG14 regulated KLF4 expression by acting as a miR-214-3p sponge. Furthermore, miR-214-3p overexpression alleviated MPP^+^-stimulated damage in SK-N-SH cells by downregulating KLF4.

**Conclusion:**

Our current study first demonstrated the protective effect of SNHG14 knockdown on MPP^+^-stimulated cytotoxicity in SK-N-SH cells at least partially by targeting the miR-214-3p/KLF4 axis, illuminating a promising target for PD intervention and treatment.

## Highlights

- SNHG14 sequestered miR-214-3p via binding to miR-214-3p in SK-N-SH cells.
- SNHG14 regulated KLF4 expression by acting as a sponge of miR-214-3p.
- SNHG14 knockdown ameliorated MPP^+^-stimulated damage in SK-N-SH cells through regulating the miR-214-3p/KLF4 axis.

## Introduction

Parkinson’s disease (PD) is a degenerative disorder and the second common neurodegenerative disease (Barnett, 2016). PD has a high incidence with an estimated 0.1–0.2% in unselected populations, 1% in people above 60 years of age, and 4% in people more than 80 years worldwide (Tysnes and Storstein, 2017). Despite decades of research, effective treatment for PD is still limited (Waldthaler and Timmermann, 2019). A better understanding of what drives PD pathology is of particular importance for developing innovative therapeutic interventions.

Long non-coding RNAs (lncRNAs) are longer than 200 nucleotides transcripts that are emerging as diverse regulators in development and disease programs (Kopp and Mendell, 2018). Thousands of lncRNAs have been shed light on their critical roles on gene expression modulation through sequestering available microRNAs (miRNAs), illuminating the importance of such interactions in human diseases, including PD (Wu et al., 2013; Kraus et al., 2017; Lekka and Hall, 2018). For instance, Peng et al. (2019) identified that HAGLR opposite strand lncRNA (HAGLROS) expression was elevated in PD mouse and cell models, and negatively regulated 1-methyl-4-phenylpyridinium (MPP^+^)-evoked cell autophagy and apoptosis via mediating autophagy related 10 (ATG10) expression by sponging miR-100. Cao et al. (2018) ascertained that small nucleolar RNA host (SNHG) gene 1 (SNHG1) level was overexpressed in PD, and the silencing of SNHG1 protected microglia against lipopolysaccharide-evoked inflammatory injury by the regulation of miR-7/nod-like receptor protein 3 (NLRP3) axis. Lin et al. (2019) reported that HOX transcript antisense RNA (HOTAIR) functioned as a sponge of miR-126-5p to contribute to PD development via protecting against RAB3A interacting protein (RAB3IP) expression inhibition. Interestingly, lncRNA SNHG gene 14 (SNHG14) was reported to be highly expressed in PD, and its deficiency performed a protective effect on PD neurotoxicity, highlighting a promising target for PD treatment (Zhang et al., 2019). Nevertheless, the functional mechanisms of SNHG14 on PD pathology remain largely unknown.

Numerous researches have underscored that the deregulation of miRNAs is implicated in PD etiology (Mushtaq et al., 2016). A low serum level of miR-214 was reported as a potential biomarker for PD early diagnosis (Dong et al., 2016; Ma et al., 2016), and miR-214-3p was prominently differentially expressed in PD blood compared with normal controls using meta-analysis (Schulz et al., 2019). Moreover, the downregulation of miR-214 was associated with PD pathogenesis by targeting α-synuclein (Wang et al., 2015). However, it is not unclear whether miR-214-3p was involved in SNHG14-mediated regulation in PD pathology.

For these reasons, we undertook to identify the biological role and mechanism played by SNHG14 in PD pathology, with the hope that SNHG14 might serve as a potential target for PD intervention and treatment.

## Materials and Methods

### Animal and Treatment

Animal experimental protocols in this study were approved by the Ethics Committee of Huaihe Hospital, Henan University, and all procedures were in line with the National Guidance of the Care and Use of Laboratory Animals. Twelve male C57BL/6 mice (10 weeks of age; Guangdong Medical Laboratory Animal Center, Guangdong, China) were maintained on a specific-pathogen-free environment and used for the establishment of 1-methyl-4-phenyl-1,2,3,6-tetrahydropyridine (MPTP) PD mouse models as reported (Cao et al., 2018). All mice were divided into two groups (*n* = 6 per group): (1) MPTP group, intraperitoneal injection of MPTP-HCl (30 mg/kg; Meilunbio, Dalian, China) every day for 7 consecutive days; (2) control group, injection of the same volume of PBS in the same way. Three days after the last injection, these mice were sacrificed, and the ventral midbrain tissues were collected and stored in liquid nitrogen until use.

### Primary Neuron Preparation, Cell Culture, and Treatment

The cortices from C57BL/6 embryos (Guangdong Medical Laboratory Animal Center) were stripped out and were digested in 0.025% trypsin (Invitrogen, Paisley, Scotland, United Kingdom). The supernatant was plated in culture dishes; maintained in neurobasal medium; enriched with B27 supplement, 1% penicillin/streptomycin, and 1 × glutamax (all from Invitrogen); and cultured for 7 days for further experiments. Primary cells at ∼70% confluence were stimulated with MPP^+^ (Meilunbio) at different final concentrations of 0.5, 1, and 1.5 mM for 24 h in an atmosphere of 5% CO_2_ at 37°C.

SK-N-SH cells (ATCC HTB-11; ATCC, Manassas, VA, United States) were maintained using standard protocols (composed of Eagle’s Minimum Essential Medium and 10% fetal bovine serum) provided by the ATCC. The cells at ∼70% confluence were stimulated with 1 mM of MPP^+^ for 24 h at 37°C.

### Quantitative Real-Time Polymerase Chain Reaction (qRT-PCR)

TRIzol reagent (Invitrogen) was used for RNA preparation from midbrain tissues and cells following the manufacturer’s recommendations. The expression levels of SNHG14 and Krüppel-like factor 4 (KLF4) were gauged using PrimeScript One Step TB Green RT-PCR Kit (TaKaRa, Beijing, China) in 25 μl of reaction containing 200 ng of total RNA with the specific primers sets (provided in Table 1; BGI, Shenzhen, China). The quantification of miR-214-3p was performed using TaKaRa miRNA qRT-PCR TB Green Kit and primers specific for miR-214-3p (provided in Table 1; BGI). The Cycle Dice System (TaKaRa) was used for all reactions as recommended by the manufacturers. The relative levels of SNHG14, KLF4, and miR-214-3p were calculated by the 2^–ΔΔ*Ct*^ method (Cao et al., 2018) and normalized to β-actin or U6.

**TABLE 1:** Primer sequences used for PCR.

Primers for PCR (5′–3′)		
SNHG14-Human	Forward	GGGTGTTTACGTAGACCAGAACC
	Reverse	CTTCCAAAAGCCTTCTGCCTTAG
SNHG14-Mouse	Forward	ACCTGCAAGCTTTTTGACCC
	Reverse	AGCAGACAAAGAAAAACCCCAAT
KLF4-Human	Reverse	CTTCCAAAAGCCTTCTGCCTTAG
	Reverse	CCACAGCCGTCCCAGTCACAGTGG
β-actin-Human	Forward	CATGTACGTTGCTATCCAGGC
	Reverse	CGCTCGGTGAGGATCTTCATG
β-actin-Mouse	Forward	CCAACCGTGAAAAGATGACC
	Reverse	CCAGAGGCATACAGGGACAG
miR-214-3p-Human	Forward	GTGCAGGGTCCGAGGT
	Reverse	ATCATAGAGGAAAATCCACG
miR-214-3p-Mouse	Forward	GTCCGCACAGCAGGCACAGACAGGCAGT
	Reverse	GTGCGTGTCGTGGAGTC
U6-Human	Forward	CTCGCTTCGGCAGCACA
	Reverse	AACGCTTCACGAATTTGCGT
U6-Mouse	Forward	GTGATCACTCCCTGCCTGAG
	Reverse	GGACTTCACTGGACCAGACG

### Cell Transfection

siRNA for SNHG14 (si-SNHG14, 5′-GCACAAUAUCUUU GAACUA-3′), siRNA for KLF4 (si-KLF4, 5′-AUUAUCC ACUCACAAGAUGAC-3′) and negative siRNA control (si-NC, 5′-UUCUCCGAACGUGUCACGUAU-3′), pcDNA-based recombinant plasmids SNHG14 and KLF4 and non-target pcDNA plasmid, miR-214-3p mimic (5′-UGACGGACAGACACGGACGACA-3′) and scrambled mimic control (5′-ACUCUAUCUGCACGCUGACUU-3), and miR-214-3p inhibitor (anti-miR-214-3p, 5′-UGUCGUCC GUGUCUGUCCGUCA-3′) and a corresponding negative sequence (anti-miR-NC, 5′-UGACUGUACUGAACUCGACUG-3′) were obtained from GenePharma (Shanghai, China) and individually transfected into SK-N-SH cells using Lipofectamine 3000 (Invitrogen) based on the instructions of the manufacturers.

### Cell Viability and Apoptosis Assays

SK-N-SH cells of ∼70% confluence were transfected with the indicated oligonucleotides or plasmids and then treated with 1 mM of MPP^+^ for 24 h. The sensitive colorimetric assay for cell viability was carried out using Cell Counting-8 Kit (CCK-8; Abcam, Cambridge, United Kingdom) as recommended by the manufacturers. The flow cytometry for cell apoptosis was carried out using the double staining of fluorescein isothiocyanate–tagged Annexin V and propidium iodide (PI; Elabscience, Wuhan, China) based on the manufacturer’s guidance.

### ELISA

Measurement of tumor necrosis factor-α (TNF-α), interleukin-6 (IL-6), and IL-1β levels were done using Human ELISA Kits (Abcam) following the directions of the manufacturers.

### Bioinformatics and Dual-Luciferase Reporter Assay

The computational software starBase version 2.0^1^ was used to search the targeted miRNAs of SNHG14 and miR-214-3p-binding sites. SNHG14 wild-type reporter vector (WT-SNHG14) harboring the complementary sites for miR-214-3p, KLF4 3U′TR wild-type reporter vector (KLF4 3′UTR-WT), and the two corresponding mutants (MUT-SNHG14 and KLF4 3′UTR-MUT) in the target region were constructed by R&S Biotechnology (Shanghai, China). SK-N-SH cells were transfected with WT-SNHG14, MUT-SNHG14, KLF4 3′UTR-WT, or KLF4 3′UTR-MUT together with miR-214-3p mimic or miR-NC mimic. The cells were harvested 24 h post-transfection, and luciferase activity was analyzed using the Dual-luciferase Assay System (Promega, Southampton, United Kingdom).

### RNA Immunoprecipitation (RIP) Assay

Cell lysates were prepared using the Pierce IP Lysis buffer (Thermo Fisher Scientific, Hemel Hempstead, United Kingdom) and subsequently incubated with antibody against Argonaute 2 (Ago2, MA5-23515; Invitrogen) or isotype IgG (ab200699; Abcam) for 3 h at 4°C, followed by the addition of magnetic A/G beads for 3 h. Beads were harvested, and total RNA was extracted. The levels of SNHG14, miR-214-3p, and KLF4 were gauged by qRT-PCR.

### Western Blot for KLF4 Expression

Total protein of midbrain tissues and cells was obtained using Invitrogen Lysis buffer and quantified by the BCA method (Thermo Fisher Scientific). An equal amount of protein (50 μg) was subjected to electrophoresis on 10% SDS-polyacrylamide gels and then blotted on nitrocellulose membranes (Thermo Fisher Scientific). After being blocked in 5% non-fat milk, the membranes were probed with anti-KLF4 (ab129473; Abcam) or anti-glyceraldehyde-3-phosphate dehydrogenase (anti-GAPDH, ab9485; Abcam) antibody, followed by the addition of horseradish peroxidase-conjugated secondary antibody (ab205718; Abcam). Immunoblots were developed by the enhanced chemiluminescence method (Thermo Fisher Scientific) as recommended by the manufacturers.

### Statistical Analysis

Data were presented as the mean ± standard deviation. Student’s *t*-test (two-tailed) or ANOVA was used for the determination of statistical significance. *P*-values at 0.05 or smaller were considered significant.

## Results

### SNHG14 Was Upregulated and MiR-214-3p Was Downregulated in PD Models

**FIGURE 1:**
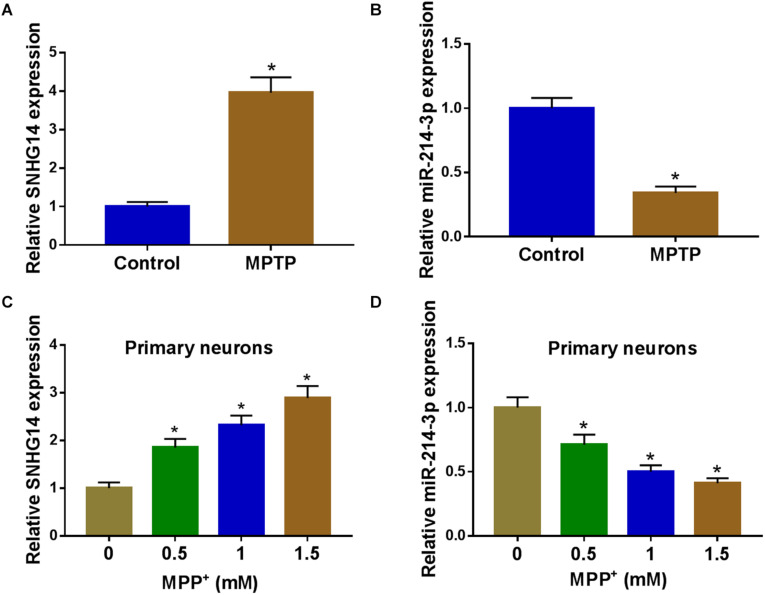
SNHG14 was upregulated and miR-214-3p was downregulated in PD mouse and cell models. SNHG14 and miR-214-3p levels were assessed by qRT-PCR in six MPTP-treated mice and six normal controls **(A,B)**, and primary neuronal cells after treatment with the indicated concentrations of MPP^+^ for 24 h **(C,D)**. *P < 0.05.

To preliminarily observe the involvement of SNHG14 and miR-214-3p in PD pathology, we first established the *in vivo* and *in vitro* models of PD by MPTP-mediated mouse and MPP^+^-stimulated primary neuronal cells. As shown by qRT-PCR, SNHG14 expression was significantly increased and miR-214-3p level was remarkably decreased in MPTP-mediated PD mouse (Figures 1A,B). Moreover, MPP^+^ stimulation elevated SNHG14 level and reduced miR-214-3p expression in a dose-dependent manner in primary neuronal cells (Figures 1C,D).

### SNHG14 Knockdown Ameliorated MPP^+^-Stimulated SK-N-SH Cell Damage

**FIGURE 2:**
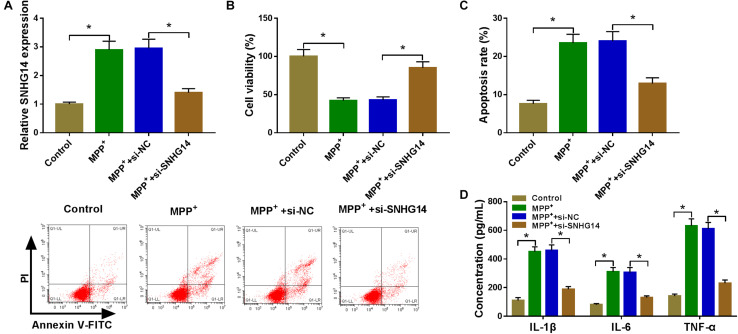
SNHG14 knockdown protected SK-N-SH cell against MPP^+^-stimulated cytotoxicity. SK-N-SH cells were transfected with or without si-NC or si-SNHG14 and then exposed to 1 mM of MPP^+^ for 24 h. **(A)** SNHG14 expression was detected by qRT-PCR in treated cells. **(B)** Cell viability was assessed by CCK-8 assay. **(C)** Cell apoptosis was determined by flow cytometry. **(D)** The levels of IL-1β, IL-6, and TNF-α were evaluated by ELISA using human assay kits. *P < 0.05.

To determine the role of SNHG14 in PD pathology, we performed “phenocopy” silencing by si-SNHG14. Transient introduction of si-SNHG14, but not the si-NC control, prominently reduced SNHG14 expression in MPP^+^-stimulated SK-N-SH cells (Figure 2A). The results of CCK-8 and flow cytometry assays showed that in contrast to the normal control, the stimulation of MPP^+^ led to a significant reduction in cell viability (Figure 2B) and a striking promotion in cell apoptosis (Figure 2C). MPP^+^ stimulation also resulted in increased levels of IL-1β, IL-6, and TNF-α in SK-N-SH cells (Figure 2D), indicating the enhancement of MPP^+^ on cell inflammation. Subsequently, in comparison with the control group, the knockdown of SNHG14 caused a strong enhancement in cell viability (Figure 2B), a remarkable repression in cell apoptosis (Figure 2C), as well as a distinct reduction in cell inflammation (Figure 2D) in MPP^+^-stimulated SK-N-SH cells. In addition, our data showed that SNHG14 overexpression significantly repressed cell viability when comparing with the negative control in MPP^+^-treated SK-N-SH cells (Supplementary Figure 1A).

### SNHG14 Sequestered MiR-214-3p via Binding to MiR-214-3p in SK-N-SH Cells

**FIGURE 3:**
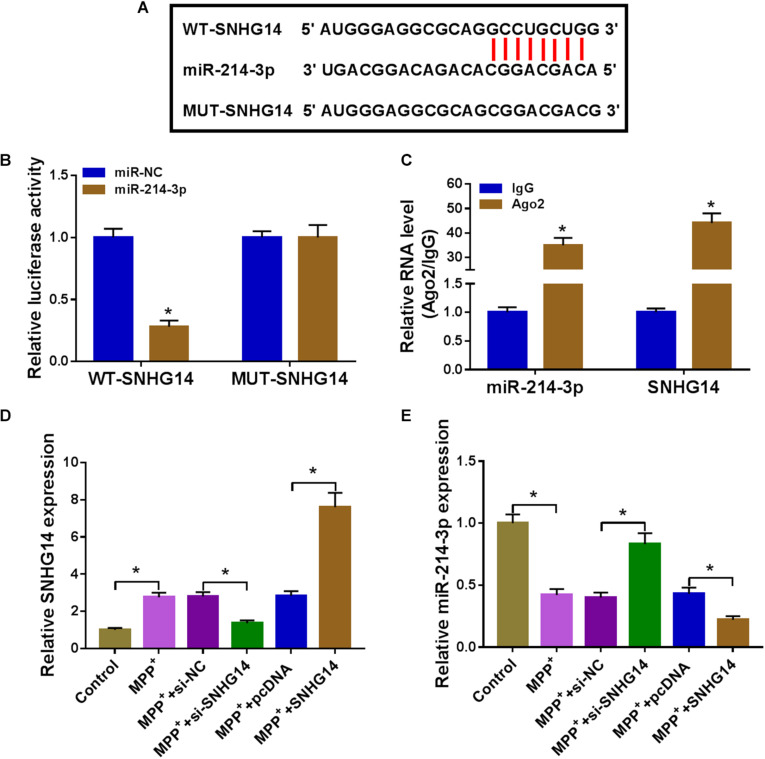
SNHG14 sequestered miR-214-3p via binding to miR-214-3p. **(A)** Schematic of the miR-214-3p seed sites within SNHG14 identified by the starBase software and mutated the seed sites. **(B)** Luciferase assays were done in SK-N-SH cells cotransfected with WT-SNHG14 or MUT-SNHG14 and miR-214-3p mimic or miR-NC mimic. **(C)** RIP assays were performed using anti-Ago2 or anti-IgG antibody in cell lysates of SK-N-SH cells. **(D,E)** SK-N-SH cells were transfected with si-NC, si-SNHG14, pcDNA, or SNHG14 overexpression vector before MPP^+^ (1 mM, 24 h) stimulation, followed by the measurement of SNHG14 and miR-214-3p levels by qRT-PCR. pcDNA: negative control plasmid, SNHG14: SNHG14 overexpression vector. *P < 0.05.

We then determined the mechanism by which SNHG14 regulated MPP^+^-stimulated cytotoxicity. Computer algorithms predicted that SNHG14 harbors eight nucleotides that match the seed sequence of miR-214-3p (Figure 3A). To verify this finding, we carried out luciferase activity assays using SNHG14 wild-type reporter (WT-SNHG14). With WT-SNHG14 and miR-214-3p, overexpression triggered a remarkable reduction in luciferase activity (Figure 3B). To validate whether the miR-214-3p-binding sites were required for this effect, a mutant SNHG14 reporter (MUT-SNHG14), in which all eight predicted complementary sites were mutated, was tested. Notably, this mutant no longer elicited such an effect (Figure 3B). Ago2 is the core component of the RNA-induced silencing complex (RISC), where miRNAs silence gene expression (Golden et al., 2017). Hence, we performed RIP experiments using anti-Ago2 antibody. These results revealed that in contrast to the negative control, the levels of SNHG14 and miR-214-3p were synchronously elevated by anti-Ago2 antibody (Figure 3C), demonstrating their potential endogenous interplay in SK-N-SH cells. The data of qRT-PCR showed that the introduction of SNHG14 overexpression vector caused a significant augment in SNHG14 expression in SK-N-SH cells (Figure 3D). Furthermore, in comparison with their counterparts, miR-214-3p expression was remarkably increased by SNHG14 knockdown, and it was markedly decreased by the upregulation of SNHG14 (Figure 3E).

### MiR-214-3p Mediated the Protective Effect of SNHG14 Knockdown on MPP^+^-Stimulated SK-N-SH Cell Damage

**FIGURE 4:**
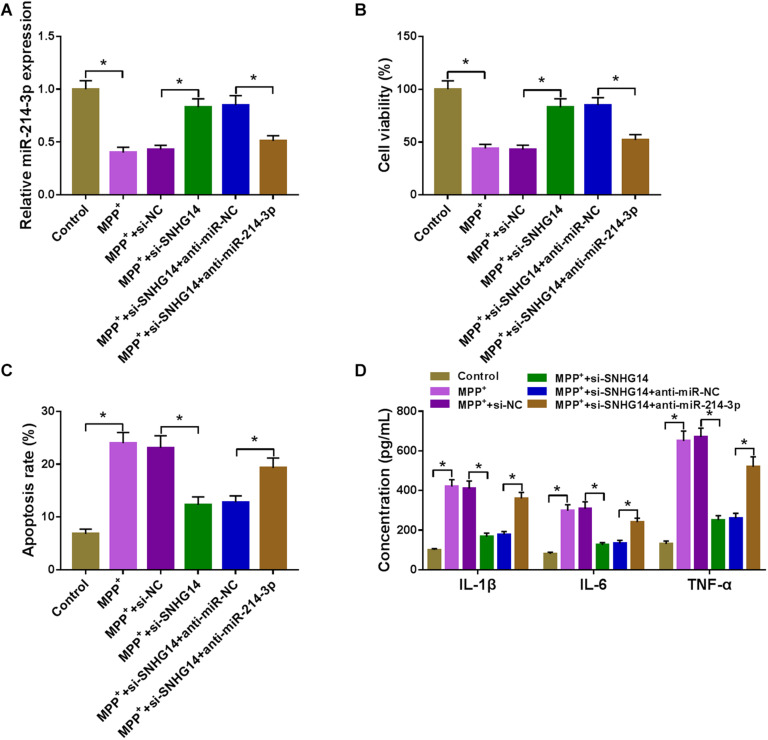
SNHG14 knockdown alleviated MPP^+^-stimulated SK-N-SH cell injury by upregulating miR-214-3p. MiR-214-3p expression was gauged by qRT-PCR **(A)**; cell viability was assessed by CCK-8 assay **(B)**; cell apoptosis was determined by flow cytometry **(C)**; the levels of IL-1β, IL-6, and TNF-α were evaluated by ELISA **(D)** in MPP^+^-stimulated SK-N-SH cells transfected with si-NC, si-SNHG14, si-SNHG14 + anti-miR-NC, or si-SNHG14 + anti-miR-214-3p. *P < 0.05.

Next, we examined whether SNHG14 regulated MPP^+^-stimulated cytotoxicity by miR-214-3p. In contrast to the control group, si-SNHG14-mediated miR-214-3p upregulation was significantly abolished by anti-miR-214-3p introduction in MPP^+^-stimulated SK-N-SH cells (Figure 4A). Moreover, si-SNHG14-induced pro-viability (Figure 4B), anti-apoptosis (Figure 4C), and anti-inflammation (Figure 4D) effects were remarkably reversed by the restored expression of miR-214-3p in MPP^+^-stimulated SK-N-SH cells.

### MiR-214-3p Directly Interacted With the 3′UTR of KLF4

**FIGURE 5:**
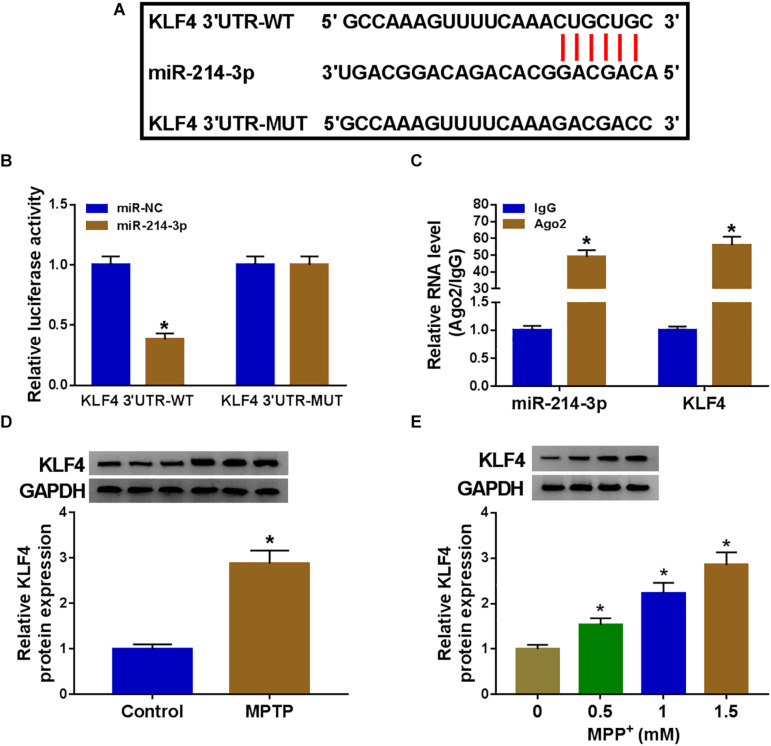
MiR-214-3p directly interacted with the 3′UTR of KLF4. **(A)** Schematic of the putative target sequence for miR-214-3p within KLF4 3′UTR and the mutant in the target sequence. **(B)** Luciferase activities were evaluated in SK-N-SH cells transfected with KLF4 3′UTR-WT or KLF4 3′UTR-MUT together with miR-NC mimic or miR-214-3p mimic. **(C)** The enrichment levels of miR-214-3p and KLF4 by anti-Ago2 or anti-IgG antibody in cell lysates of SK-N-SK cells. **(D,E)** KLF4 protein expression was tested by western blot in 6 MPTP-treated mice and 6 normal controls, and primary neuronal cells after treatment with the indicated concentrations of MPP^+^ for 24 h. *P < 0.05.

The potential targets of miR-214-3p were predicted by the starBase software, and the data revealed a putative target sequence (CUGCUG) for miR-214-3p within KLF4 3′UTR (Figure 5A). To confirm this, KLF4 3′UTR wild-type reporter (KLF4 3′UTR-WT) or KLF4 3′UTR mutant-type construct (KLF4 3′UTR-MUT) was transfected into SK-N-SH cells. In contrast to the control group, the transfection of miR-214-3p mimic dramatically reduced the luciferase activity of KLF4 3′UTR-WT but barely affected the luciferase of the mutant (Figure 5B), demonstrating the validity of the target sequence for interaction. Moreover, miR-214-3p and KLF4 were concurrently enriched by anti-Ago2 antibody in SK-N-SH cells (Figure 5C). In addition, in comparison with the corresponding negative control, KLF4 protein level was prominently increased in MPTP-mediated PD mouse and MPP^+^-stimulated primary neuronal cells (Figures 5D,E).

### MiR-214-3p Overexpression Alleviated MPP^+^-Stimulated SK-N-SH Cell Damage by Downregulating KLF4

**FIGURE 6:**
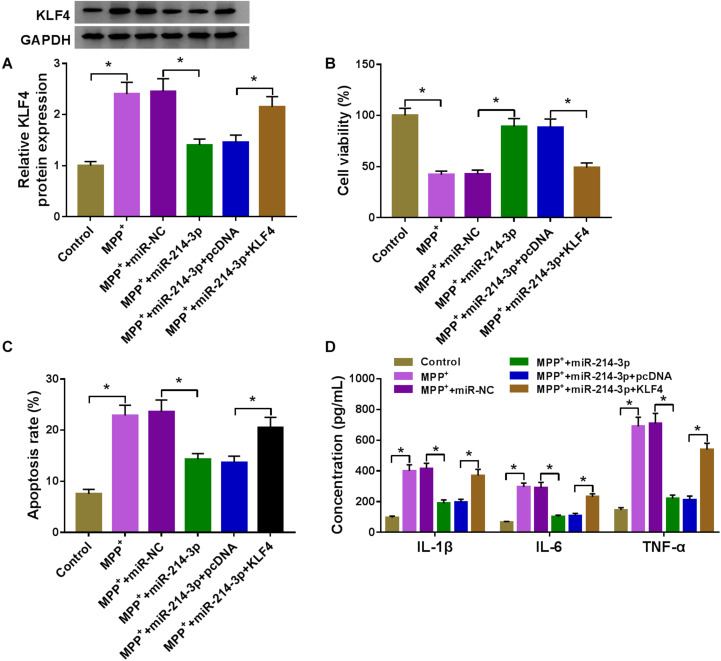
The overexpression of miR-214-3p alleviated MPP^+^-stimulated SK-N-SH cell injury by downregulating KLF4. SK-N-SH cells were transfected with miR-NC mimic, miR-214-3p mimic, miR-214-3p mimic + pcDNA, or miR-214-3p mimic + KLF4 overexpression vector before MPP^+^ (1 mM, 24 h) treatment, followed by the detection of KLF4 protein expression by western blot **(A)**, cell viability by CCK-8 assay **(B)**, cell apoptosis by flow cytometry **(C)**, the levels of IL-1β, IL-6, and TNF-α by ELISA **(D)**. pcDNA: negative control plasmid, KLF4: KLF4 overexpression vector. *P < 0.05.

As demonstrated by western blot, in comparison with the miR-NC control, miR-214-3p upregulation resulted in decreased expression of KLF4 protein (Figure 6A), indicating that KLF4 was a direct target of miR-214-3p in MPP^+^-stimulated SK-N-SH cells. Functional analyses data showed that the overexpression of miR-214-3p led to a striking enhancement in cell viability (Figure 6B), a prominent suppression in cell apoptosis (Figure 6C), as well as a strong reduction in IL-1β, IL-6, and TNF-α production (Figure 6D) in MPP^+^-stimulated SK-N-SH cells. These data together pointed that the upregulation of miR-214-3p alleviated MPP^+^-stimulated cytotoxicity. Besides, by contrast, the data of CCK-8 revealed that miR-214-3p knockdown remarkably suppressed cell viability (Supplementary Figure 1B).

The transfection efficiency of si-KLF4 was confirmed by western blot, and CCK-8 assay revealed that KLF4 knockdown led to a significant promotion in cell viability in MPP^+^-treated SK-N-SH cells (Supplementary Figures 1C,D). To provide further mechanistic sight into the relationship between miR-214-3p and KLF4 on MPP^+^-stimulated cytotoxicity, miR-214-3p mimic and KLF4 overexpression vector were cotransfected into SK-N-SH cells before MPP^+^ treatment. In comparison with the negative control, miR-214-3p overexpression-mediated KLF4 downregulation was notably abolished by KLF4 overexpression vector (Figure 6A). Furthermore, miR-214-3p overexpression-induced pro-viability (Figure 6B), anti-apoptosis (Figure 6C), and anti-inflammation (Figure 6D) effects were strikingly abrogated by KLF4 expression restoration in MPP^+^-treated SK-N-SH cells.

### SNHG14 Regulated KLF4 Expression by Acting as a MiR-214-3p Sponge

**FIGURE 7:**
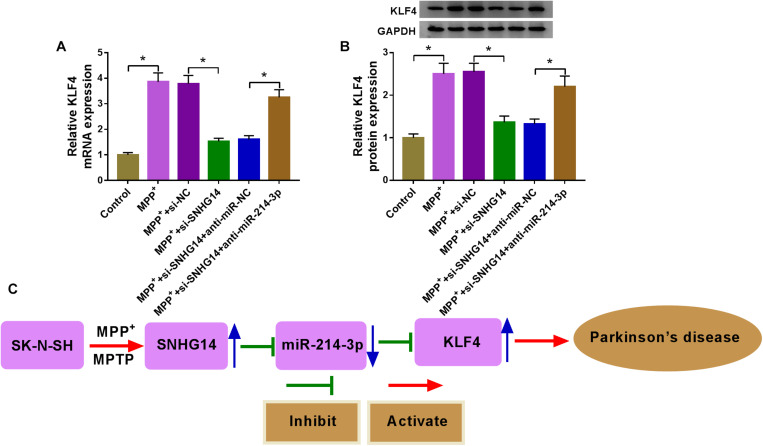
SNHG14 regulated KLF4 expression by acting as a miR-214-3p sponge. **(A,B)** SK-N-SH cells were transfected with si-NC, si-SNHG14, si-SNHG14 + anti-miR-NC, or si-SNHG14 + anti-miR-214-3p before MPP^+^ (1 mM, 24 h) stimulation, and then KLF4 mRNA and protein levels were gauged by qRT-PCR and western blot assays. *P < 0.05. **(C)** Schematic model of the SNHG14/miR-214-3p/KLF4 axis in MPP^+^-stimulated cytotoxicity in PD. MPP^+^ stimulation elevated the expression of SNHG14 in SK-N-SH cells. Then, the elevated expression of SNHG14 downregulated miR-214-3p expression, and thus upregulated KLF4 level in MPP^+^-stimulated SK-N-SH cells. Finally, the overexpression of KLF4 accelerated MPP^+^-stimulated cytotoxicity in PD.

A key question was whether SNHG14 could regulate KLF4 expression. The data of qRT-PCR and western blot revealed that MPP^+^ treatment triggered a significant upregulation in KLF4 expression at both mRNA and protein levels (Figures 7A,B). Moreover, as expected, KLF4 mRNA and protein levels were remarkably reduced by SNHG14 silencing in MPP^+^-treated SK-N-SH cells, and these effects were prominently abolished by the cotransfection of anti-miR-214-3p in MPP^+^-stimulated SK-N-SH cells (Figures 7A,B).

## Discussion

PD is a common age-related neurodegenerative disease, leading to impaired quality of life (Beitz, 2014). Owing to the inadequate knowledge in PD pathology, current treatments focus on symptom mitigation instead of PD prevention and fundamental treatment (Ng, 2018). LncRNAs are emerging as key regulators in PD pathogenesis, and the identification of their function remains a major challenge (Majidinia et al., 2016; Riva et al., 2016). In the present work, our data supported that SNHG14 knockdown protected against MPP^+^-stimulated cytotoxicity, as has been reported (Zhang et al., 2019). Mechanistically, SNHG14 knockdown alleviated MPP^+^-induced PD progression *in vitro* via the regulation of the miR-214-3p/KLF4 axis.

Recent studies indicated that SNHG14 functioned as a tumor promoter by sponging different miRNAs in a series of cancers, such as retinoblastoma, pancreatic ductal adenocarcinoma, and osteosarcoma (Hou and Mao, 2020; Sun et al., 2020; Xie et al., 2020). SNHG14 was uncovered to be highly expressed in ischemic stroke, and the inhibition of SNHG14 mitigated neuron inflammatory damage (Qi et al., 2017; Zhong et al., 2019). In addition, the silencing of SNHG14 was reported to relieve LPS-triggered inflammation in the model of acute lung injury by targeting the miR-34c-3p/WISP1 axis (Zhu et al., 2019). Here, we demonstrated a significant overexpression of SNHG14 in PD mice and cells, in line with the findings by Zhang et al. (2019). We also substantiated that the knockdown of SNHG14 ameliorated MPP^+^-stimulated damage in SK-N-SH cells, as evidenced by the enhancement in cell viability and the suppression in cell apoptosis and pro-inflammatory cytokine production.

LncRNAs can fine-tune gene expression through acting as miRNA sponges to sequester available miRNAs. Here, we were first to demonstrate that SNHG14 targeted miR-214-3p via directly binding to miR-214-3p in SK-N-SH cells. Among thousands of candidates predicted by the starBase software, miR-214-3p was fascinating because of its key role in multitudinous human diseases. For example, miR-214-3p regulated the tumorigenesis of ovarian cancer, endometrial cancer, and papillary thyroid carcinoma (Fang et al., 2019; Yang C. et al., 2019; Sui et al., 2020). MiR-214-3p was identified to repress cardiac fibrosis through the inhibition of the NOD-like receptor family CARD domain containing 5 (Yang K. et al., 2019). In addition, miR-214-3p expression was downregulated in the rat model of spinal nerve ligation, and the elevated miR-214-3p expression mitigated neuroinflammation and neuropathic pain by targeting colony-stimulating factor-1 (Yang K. et al., 2019). MiR-214-3p also associated with the neuroprotection of Sigma 1 receptor in retinitis pigmentosa (Wang and Smith, 2019). We subsequently uncovered a protective effect of miR-214-3p overexpression on MPP^+^-stimulated cytotoxicity. More interestingly, for the first time, we identified that SNHG14 knockdown mitigated MPP^+^-stimulated cytotoxicity in SK-N-SH cells by upregulating miR-214-3p.

Here, we first confirmed that KLF4 in SK-N-SH cells was directly targeted and suppressed by miR-214-3p. KLF4, a transcription factor and cell-cycle suppressor, has been shown to be implicated in various human diseases (Wang et al., 2016, 2018; Xu et al., 2020). In addition, KLF4 was uncovered to be relevant to the pathogenesis of Alzheimer’s disease (Cheng et al., 2018). Previous evidence also reported that KLF4 silencing protected against MPP^+^-evoked cytotoxicity in PD (Chen et al., 2013; Kong et al., 2016). In this research, we were first to demonstrate that miR-214-3p overexpression alleviated MPP^+^-evoked cytotoxicity via targeting KLF4. Similarly, Kong et al. (2016) confirmed that miR-7 protected SH-SY5Y cell from MPP^+^-triggered apoptosis by targeting KLF4. Song et al. (2018) uncovered that miR-212 directly targeted KLF4 to relieve MPP^+^-stimulated cell injury. More interestingly, we first uncovered that SNHG14 modulated KLF4 expression via acting as a sponge of miR-214-3p.

In conclusion, the current study provided evidence for the protective role of SNHG14 knockdown on MPP^+^-stimulated cytotoxicity in SK-N-SH cells through targeting the miR-214-3p/KLF4 axis. It is the first research for the SNHG14/miR-214-3p/KLF4 regulatory network in PD pathogenesis (Figure 7C), illuminating a promising target for intervention and treatment.

## Data Availability Statement

The raw data supporting the conclusions of this article will be made available by the authors, without undue reservation.

## Ethics Statement

The animal experimental protocols in this study were approved by the Ethics Committee of Huaihe Hospital, Henan University and all procedures were in line with the National Guidance of the Care and Use of Laboratory Animals.

## Author Contributions

SZ and DZ conceived and designed the experiments. JG performed the experiments and funding acquisition. JZ contributed reagents, materials, and analysis tools. YC wrote the article. All authors contributed to the article and approved the submitted version.

## Conflict of Interest

The authors declare that the research was conducted in the absence of any commercial or financial relationships that could be construed as a potential conflict of interest.

## Footnotes

1 http://starbase.sysu.edu.cn/

## Supplementary Material

FIGURE S1 The effects of SNHG14 overexpression, miR-214-3p knockdown, or KLF4 knockdown on SK-N-SH cell viability under MPP^+^ stimulation. **(A)** CCK-8 assay for cell viability in MPP^+^-stimulated SK-N-SH cells transfected with pcDNA or SNHG14. pcDNA: negative control plasmid, SNHG14: SNHG14 overexpression vector. **(B)** Cell viability by CCK-8 assay in MPP^+^-stimulated SK-N-SH cells transfected with anti-miR-NC or anti-miR-214-3p. KLF4 protein level by western blot **(C)** and cell viability by CCK-8 assay **(D)** in MPP^+^-stimulated SK-N-SH cells transfected with si-NC or si-KLF4. ^∗^*P* < 0.05.
