# Supplementary figures and images for: Knockdown of SNHG14 Alleviates MPP+-Induced Injury in the Cell Model of Parkinson’s Disease by Targeting the miR-214-3p/KLF4 Axis
Source: Front Neurosci. 2020 Sep 21;14:930. doi: 10.3389/fnins.2020.00930 (PMC7536369; doi:10.3389/fnins.2020.00930)

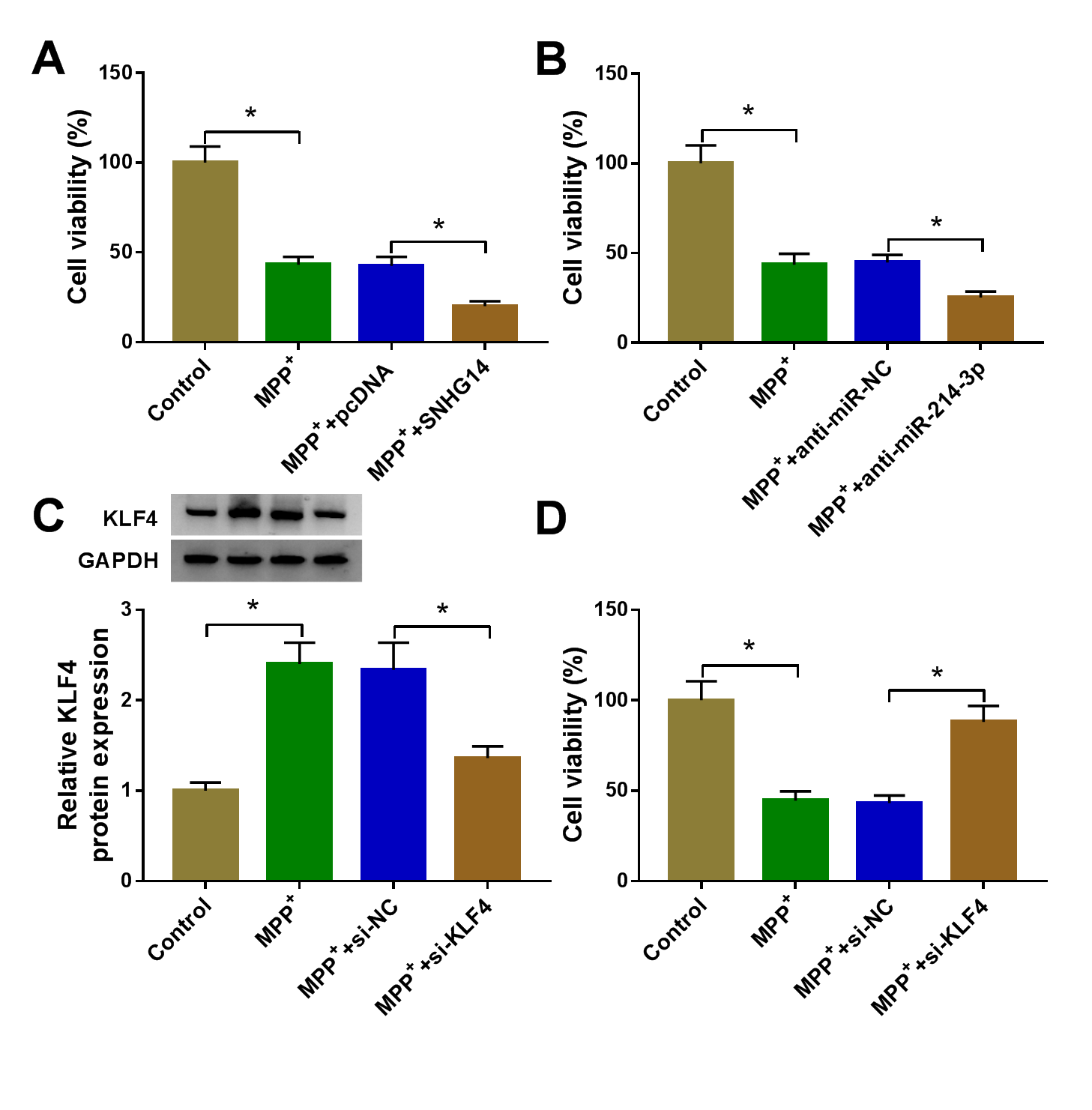

Supplement: FIGURE S1 — The effects of SNHG14 overexpression, miR-214-3p knockdown, or KLF4 knockdown on SK-N-SH cell viability under MPP+ stimulation. (A) CCK-8 assay for cell viability in MPP+-stimulated SK-N-SH cells transfected with pcDNA or SNHG14. pcDNA: negative control plasmid, SNHG14: SNHG14 overexpression vector. (B) Cell viability by CCK-8 assay in MPP+-stimulated SK-N-SH cells transfected with anti-miR-NC or anti-miR-214-3p. KLF4 protein level by western blot (C) and cell viability by CCK-8 assay (D) in MPP+-stimulated SK-N-SH cells transfected with si-NC or si-KLF4. ∗P < 0.05. [file Image_1.TIF]
